# A Beamformer Analysis of MEG Data Reveals Frontal Generators of the Musically Elicited Mismatch Negativity

**DOI:** 10.1371/journal.pone.0061296

**Published:** 2013-04-09

**Authors:** Claudia Lappe, Olaf Steinsträter, Christo Pantev

**Affiliations:** 1 Institute for Biomagnetism and Biosignalanalysis, University of Münster, Münster, Germany; 2 GSI Helmholzzentrum für Schwerionenforschung GmbH, Darmstadt, Germany; National University of Singapore, Singapore

## Abstract

To localize the neural generators of the musically elicited mismatch negativity with high temporal resolution we conducted a beamformer analysis (Synthetic Aperture Magnetometry, SAM) on magnetoencephalography (MEG) data from a previous musical mismatch study. The stimuli consisted of a six-tone melodic sequence comprising broken chords in C- and G-major. The musical sequence was presented within an oddball paradigm in which the last tone was lowered occasionally (20%) by a minor third. The beamforming analysis revealed significant right hemispheric neural activation in the superior temporal (STC), inferior frontal (IFC), superior frontal (SFC) and orbitofrontal (OFC) cortices within a time window of 100–200 ms after the occurrence of a deviant tone. IFC and SFC activation was also observed in the left hemisphere. The pronounced early right inferior frontal activation of the auditory mismatch negativity has not been shown in MEG studies so far. The activation in STC and IFC is consistent with earlier electroencephalography (EEG), optical imaging and functional magnetic resonance imaging (fMRI) studies that reveal the auditory and inferior frontal cortices as main generators of the auditory MMN. The observed right hemispheric IFC is also in line with some previous music studies showing similar activation patterns after harmonic syntactic violations. The results demonstrate that a deviant tone within a musical sequence recruits immediately a distributed neural network in frontal and prefrontal areas suggesting that top-down processes are involved when expectation violation occurs within well-known stimuli.

## Introduction

The auditory mismatch negativity is an event-related brain response elicited after an acoustic change within a repetitive regular auditory stimulation. It can be evoked after changes in frequency, intensity, timbre or duration of an acoustic stimulus as well as after changes within tone pairs or tone sequences [1]. The mismatch negativity (MMN) peaks about 120–250 ms after stimulus onset. Since the MMN is elicited even in the absence of attention to the sound stimuli, it is assumed that it reflects a pre-attentive mechanism for auditory change detection [2], [3]. It is, however, a widely adopted model that the presentation of a standard auditory stimulus leads to a learned regularity which serves as a top-down cue which is used to predict and evaluate bottom-up auditory inputs [4]–[6].

The MMN reflects electrophysiologically sound perception abilities, since the magnitude of the MMN component corresponds to behavioral auditory discrimination accuracy [7], [8]. Consequently, improvement in tone discrimination ability resulting from systematic learning leads to an increment of the MMN component. This phenomenon is also illustrated by short musical melodies that elicit an MMN after an infrequently occurring deviant musical tone. It has been shown that the musically elicited MMN is stronger in music experts [9]–[11] and that the MMN increases significantly after short-term piano training in novice players [12], [13]. Musical melodies are more complex than repetitive tone series. The melodic, rhythmic and harmonic structure of a musical phrase establishes expectations for upcoming musical events [14]. Expectancy violation within a musical context may occur because representations of musical structure and tonal progression already exist in our long-term memory [15].

The main generators of the auditory mismatch negativity after a frequency or duration change in a sequence of non-musical stimuli are generally believed to be located in the superior temporal gyrus and, to a lesser extent, in frontal areas. It has been demonstrated that the frontal-temporal scalp potential distribution of the auditory MMN can be modeled by two dipoles positioned bilaterally on the superior temporal plane [16]. Dipole modeling, however, requires prior assumptions about the locations and numbers of active sources. Distributed sources are difficult to localize with this method. Numerous EEG distributed minimum-norm solutions, fMRI and several optical imaging studies have shown sources within the superior temporal cortex contributing to the elicitation of the frequency and duration mismatch negativity [17]–[23]. Frontal contributions to the MMN component, on the other hand, have been demonstrated less often (for an overview see: Deouell et al., 2007), [24]. Some EEG, fMRI and optical imaging studies identified activation areas in inferior frontal cortex after a duration or frequency change within a repetitive tone series [18], [25]–[31]. Frontal contributions to the generation of the MMN component using a similar stimulation have, however, not been found in MEG localization studies so far [33].

Whereas the temporal neural activation assumedly reflects auditory error signal detection at the sensory level, little is known about the functional role of frontal MMN generators. Using frequency or duration changes in sequences of non-musical material it has been shown that frontal components peak slightly (8 ms–50 ms) later than temporal components suggesting that frontal activity is initiated by the auditory cortex [17], [26], [30]. In that case frontal activity could be interpreted as an area where, after change detection, an attention shift is triggered [32], [33]. This assumption is supported by studies demonstrating that an increase of the difference between standard and deviant leads to an enhanced amplitude of the MMN and P3, a component, which is generally associated with an attention switch towards the deviant event [33], [34]. However, top-down processes presumably play an important role in the generation of the auditory MMN, as seen in studies of learning-induced cortical plasticity. The MMN has recently been explained within the predictive coding framework (hierarchical inference in the brain) suggesting that sensory information from the environment are matched with top-down predictions. According to the model neurons minimize prediction errors by recurring bidirectional interaction [35], [36].

Within the musical context, Koelsch et al. (2000) have demonstrated another event related MMN like response called early right anterior negativity (ERAN) which is elicited about 150 ms after music syntactic irregularities (harmonically inappropriate chords) [37]. An equivalent current dipole (ECD) analysis of this component revealed neural activity in Broca's area and its right hemisphere homologue [38]. Since Broca's area is presumably involved in the processing of language, it has been suggested that a harmonic syntactic violation within a musical context and error detection within the syntactic property of a sentence is processed in similar brain areas, with a more left hemispheric dominance in language and a more right hemispheric dominance in music [39]. The functional role of the frontal generators that contribute to the elicitation of the auditory MMN component is, however, still not sufficiently understood [6]. Better understanding of the auditory MMN hence relies on our ability to properly localize and measure the different neural generators that contribute to it.

MEG has a high temporal resolution, and, in contrast to the delay of the hemodynamic response in functional MRI, allows measuring brain activity within a time resolution of milliseconds. Furthermore, the recorded brain magnetic fields in MEG are less distorted by the skull and scalp as compared to EEG, thus allowing a more accurate spatial resolution. MEG is therefore an adequate method for localizing activation areas and investigating the temporal resolution of auditory processing providing information about top-down and bottom-up strategies of auditory information processing. We investigated the neural generators of the musically elicited mismatch negativity using beamforming analysis. Beamforming is a method of source analysis of MEG sensor data in which a spatial filter is used to estimate the contribution of a given source location to the measured MEG sensor signal while filtering out the contributions of other sources. The advantage of this method is that it is not necessary to impose constraints on the source solution by determining the number and positions of equivalent current dipoles in advance [40], [41].

We addressed the MMN localization problem by analyzing a dataset from a previous event related potential (ERP) musical mismatch study [12] in which participants, who had no prior formal musical training, received 2 weeks of piano training (comprising 8 sessions each lasting 25 min) while their musical mismatch negativity was tested before and after the training. Pre-training data from these individuals did not show sufficient strong responses to conduct the beamforming analysis. The musical MMN was strong and reliable after short-term training, much increased compared to the pre-training levels. This robust musical MMN gave us the opportunity to perform beamforming analysis to localize in more detail the sources for the musical MMN. The interaction between pre- and post training conditions did not show significant activation areas since the data probably did not have sufficient power. A direct comparison of before and after training data to determine the relative contribution of the two generators in the learning was therefore not possible. Hence the focus of the present study was to localize the musically elicited mismatch negativity in the data obtained after short term musical training.

## Materials and Methods

### Participants

Twenty non-musicians (11 females) between 24 and 38 years of age participated in the study. Participants were right-handed as assessed by the Edinburgh Handedness Inventory [42], and none of them had a history of neurological or otological disorders. Audiological status was verified by pure tone audiometry. Informed written consent was obtained from all subjects to participate in the study. The study was approved by the Research Ethic Board of the University of Münster.

### Stimuli

**Figure 1:**
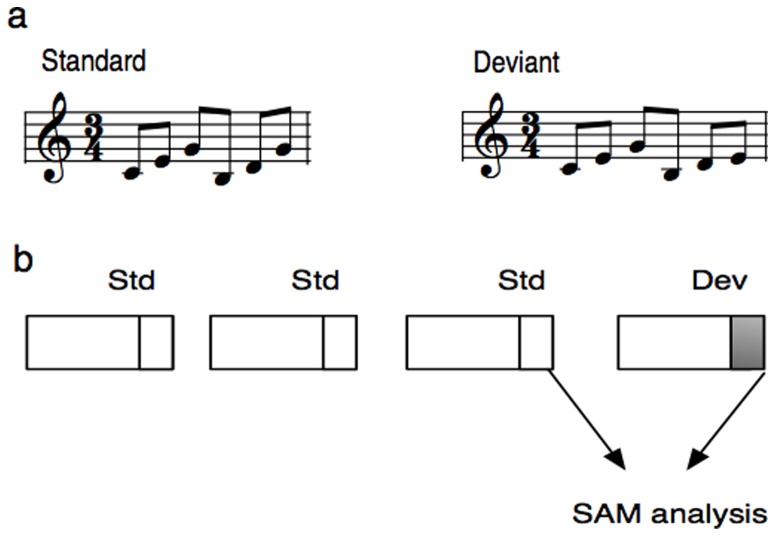
Six-tone stimulus for the MEG measurement comprising a C- and G-major broken chord. The stimuli were presented within an oddball paradigm. In the deviant stimulus the last tone was lowered by a minor third (a). A schematic diagram of the trials that were analyzed by the beamformer. Only standards preceding a deviant were included into the analysis. Deviant and standard trials were contrasted by the beamformer within two separate time windows comprising 100–200 and 200–300 ms after the onset of the last tone (b).

The stimuli for the MEG measurement consisted of a three- and a six-tone musical sequence. Since the six-tone sequence elicited the strongest MMN after training we used this stimulus for the beamformer analysis. We will therefore refer only to the data of the six-tone sequence (Fig. 1a).

The six-tone sequence of piano tones consisted of a C-major broken chord (c′-e′-g′) in root position followed by a G-major broken chord (h-d′-g′) in first inversion. The stimuli were generated by means of a digital audio workstation with an integrated on-screen virtual keyboard allowing the generation of realistic piano tones on a synthesized piano. Each tone of the piano sequence had a duration of 300 ms resulting in a total melody length of 1800 ms. The sequences were separated by a 900 ms silent interval. In the MEG measurement, the sequences were presented within an oddball paradigm in two runs, each comprising 320 standard and 80 deviant trials. On deviant trials, the last tone was lowered by a minor third. The stimuli were presented over head phones by the stimulus delivery and control software *Presentation (version 8.0,* www.neurobs.com *)*.

### MEG data acquisition

Magnetic field responses were recorded with a 275-channels whole-head magnetometer system (Omega 275; CTF Systems). As part of the data acquisition, MEG signals were low-pass filtered at 150 Hz and sampled at a rate of 600 Hz. The recordings were carried out in a magnetically shielded and acoustically silent room. Subjects were seated in an upright position as comfortably as possible, and they were instructed to move as little as possible. The subject's head position was measured at the beginning and at the end of each recording block by means of three localization coils that were fixed to the nasion and to the entrances of both ear canals (fiducial points). A silent movie was presented during the MEG measurement to distract the subjects' attention from the auditory stimulation.

A T1- weighted MR image was obtained from each participant using a three Tesla Scanner (Gyroscan Intera T30, Philips, Amsterdam, Netherlands). Turbo Field acquisition was used to collect 400 contiguous T1-weighted 0.5-mm thick slices in the sagittal plane. For co-registration with the MEG measurements the positions of the fiducial points (filled with gadolinium to be visible in the MRI) were used.

### Data analysis

Epochs contaminated by muscle or eye blink artifacts containing field amplitudes exceeding 3 pT in any channel were automatically excluded from the data analysis. For the beamformer analysis, the special beamformer approach SAM (Synthetic Aperture Magnetometry, Robinson et al. 1999) was used [43]. Epochs of 3.6 s including 0.2 s pre-stimulus intervals were extracted from the datasets and filtered by a 1–30 Hz bandpass filter. Beamformers, like any MEG analysis method, need a description of the individual electromagnetic properties of the head (sensor-weighted overlapping-sphere head model, Huang et al., 1999 [44]). Here we used the multi-sphere model fitted to the individual participants' structural MRIs as volume conductor model.

The performance of beamformers is known to degrade in the presence of two or more sources whose time courses are highly correlated with zero time lag [45], [46]. For such groups of correlated sources a beamformer, without further information about the structure of the sources, expects a single source from a single brain location. As a beamformer output is a volumetric map of the found source locations, such a signal is not represented in the output, since no single brain location is sufficient to explain the complete correlated signal [45]–[48]. However, in the main output of a classic beamformer like SAM (a volumetric map of the brain activity integrated across a given time window) only a reduction of the estimated signal strength (relative to the degree of correlation) of the sources in question must be expected. Therefore, in this paper we used SAM for the calculation of volumetric activation maps only. Nevertheless with a too high degree of correlation (with respect to the local signal-to-noise ratio), sources may fall below the significance level. To reduce this effect, at least for a time correlation between the hemispheres, we processed left temporal sensors separately from right temporal sensors, a method successfully used by Herdman et al. (2003) for the beamformer analysis of auditory signals [49]. The separate analysis of the two hemispheres resulted in a cut off down the midline hampering the interpretation of medial frontal activation. We therefore analyzed in addition the frontal channels separately.

In this study the beamformer approach SAM [43]) was used in conjunction with pseudo-T values (Robinson and Vrba, 1998). These should not be confused with statistical t-values; pseudo-T values describe the contrast in signal strength (beamformers output basically the variance of the signal of a current dipole at a given brain location across a given time window) between an “active state”, a^2^ and a “control state”, c^2^: a^2^−c^2^. To increase the depth resolution of the beamformer [50], [51], this difference was additionally normalized by an estimation of the sensor noise (singular value decomposition of the data covariance matrix) and spuriously mapped to the brain by the beamformer: n^2^. The complete pseudo-T value is therefore given as (a^2^−c^2^)/n^2^.

This technique was originally developed to separate task related activity from the background brain activity [43], but here we used this method to contrast deviant against standard. For the (a^2^−c^2^)/n^2^ calculation, the overall brain state (background activity) should be as similar as possible. We therefore contrasted each deviant with its directly preceding standard (Fig. 1b), which reduced the number of analyzed standards to the number of deviants (80; for each run and post training condition: 80×2×2 = 320 epochs).

The complete output of a beamformer calculation is a volumetric image of signal contrasts (pseudo-T values) - here with a spatial resolution of 3 mm - similar to the activation maps known from fMRI studies. For the group analysis we therefore used SPM (SPM2, software and documentation from the Wellcome Department of Imaging Neuroscience), a software package widely used in the analysis of fMRI data, for the spatial normalization of the individual beamformer output for the subjects (averaged across runs). Coordinates of brain activations are here provided in MNI coordinates.

As the assumption of normal distribution for volumetric maps of pseudo-T images is questionable [52], we could not use the parametric analysis techniques implemented with SPM but used a non-parametric permutation test method proposed by Nichols & Holmes (2001) [53] to investigate the significance of activated brain regions. This test is based on a generation of new samples of the underlying (a priori unknown) probability distribution by permuting the labels “deviant” and “control” in the measured data; as the null hypothesis assumes no statistical differences between signals connected with deviant and standard, this is allowed. The additional data are used to estimate the probability distribution and thresholds for significant brain activity that are calculated based on this distribution. Following Nichols & Holmes (2001), a maximal statistic (the maximal signal difference found for each SAM image is analyzed) is used to calculate the thresholds. This automatically takes the multiple comparison problem into account [53]. It should be noted, that statistical tests based on non-parametric methods are typically more conservative (difficult to reject the null hypothesis) than comparable parametric methods. The statistical test was done after averaging across runs and subjects. In all statistical tests, the significance level was set at 0.05.

**Figure 2:**
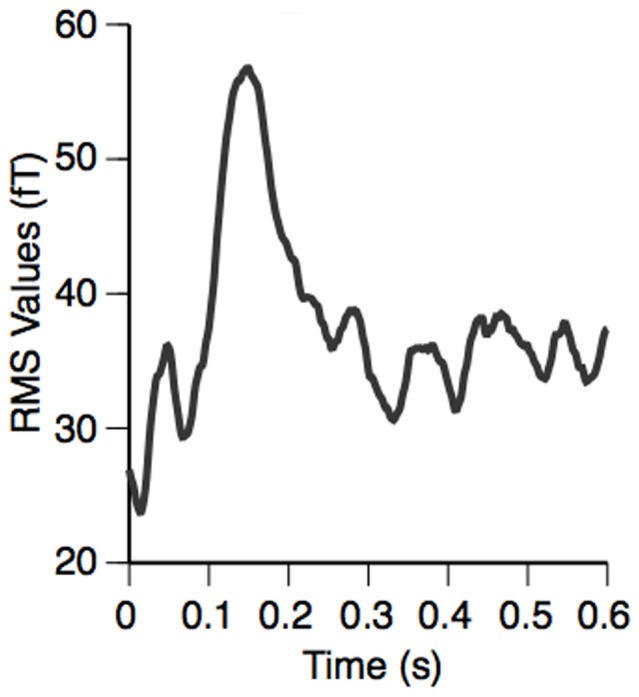
Group averages of root mean square (RMS) MEG sensor values after sensorimotor-auditory training. The MMN peak occurred around 150 ms after the onset of the deviant at time zero. Beamformer analyses were conducted within the time window of 100–200 ms, i.e. centered around the MMN, and within the time window of 200–300 ms, to look for any late components.

We conducted the beamforming analysis described above within two separate time windows. The first was centered near the peak of the musical MMN around 150 ms after the onset of the deviant tone (Fig. 2), and lasted from 100–200 ms after deviant onset. The second interval was from 200–300 ms after the occurrence of a deviant tone and served to identify any late components of the response. Both windows were contrasted to the corresponding standard time windows.

In a subsequent procedure we performed a time-course analysis using virtual channels. The coordinates of peak activation for STG and IFC in the averaged maps were back-transformed to the coordinates of each individual brain, and virtual channels were calculated based on the beamformer weights computed for the peak activation time window between 100–200 ms after onset of the deviant tone. The RMS values of the virtual channel activities were than averaged for all subjects, separately for standard and deviant trials. Then, the RMS difference between standard and deviant waveforms were determined to show the time course of the MMN activation.

## Results

**Figure 3:**
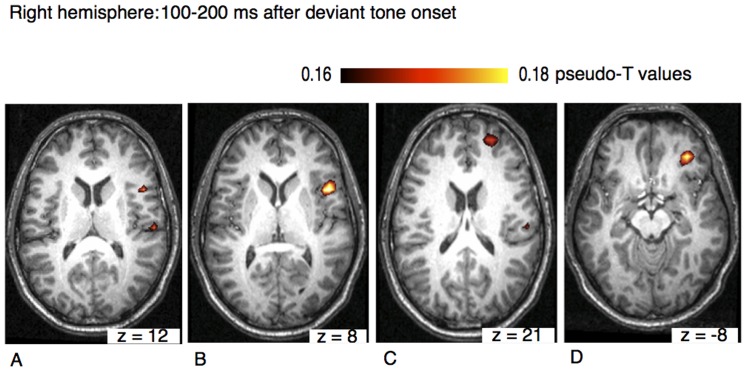
Axial view of the right hemisphere (overlaid on an individual anatomical MRI) showing significant activations (pseudo-T values, significance level 5%) of the musically elicited mismatch negativity within a time window of 100–200 ms after the occurrence of a deviant tone. Activation was found in the temporal lobe in auditory cortex (BA41) and in the inferior frontal cortex (A), in inferior frontal cortex (BA44) (B) and in the frontal lobe within the superior frontal cortex (BA10) (C). Neural activation was also found in orbitofrontal cortex (BA11) (D).

Significant neural activation was found in both time intervals and in both hemispheres. Table 1 presents a list of all activations. Neural activation was observed in the right hemisphere and in the left hemisphere. Figure 3 shows axial views of activity foci found in the right hemisphere in the time window 100–200 ms after the onset of the deviant tone. Pseudo-T values were overlaid on an individual anatomical MRI. The different panels show neural activation found in the temporal lobe in auditory cortices (BA41), in the opercular part of the inferior frontal cortex (BA44), in the superior frontal (BA10), and in the orbitofrontal cortex (BA11).

**Table 1:** Anatomical locations (MNI coordinates) of all significant activations.

Anatomical location	MNI coordinates		
Right hemisphere (100–200 ms)	x	y	z
1. Temporal lobe/auditory cortex (BA41)	62	−20	16
2. Inferior frontal cortex (BA44)	51	12	8
3. Superior frontal cortex (BA10)	26	55	21
4. Orbitofrontal cortex (BA11)	35	42	−8
Left hemisphere (100–200 ms)	x	y	z
1. Inferior frontal cortex (BA45)	51	25	10
2. Superior frontal cortex	−20	52	19
Right hemisphere (200–300 ms)	x	y	z
1. Superior frontal cortex	34	54	14
2. Superior frontal cortex	8	55	25
3. Middle frontal gyrus	36	20	34
Left hemisphere (200–300 ms)	x	y	z
1. Inferior frontal cortex	−39	37	10

**Figure 4:**
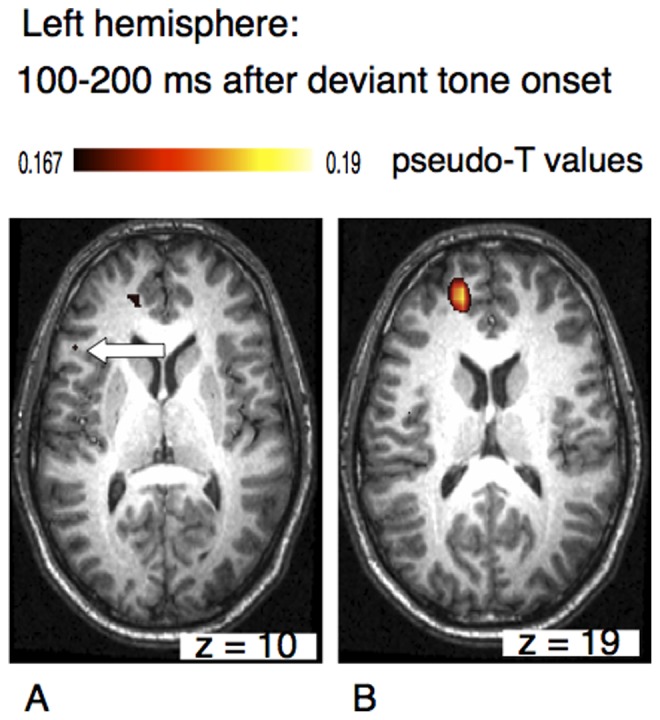
Axial view of the left hemisphere of the musically elicited mismatch negativity within a time window of 100–200 ms after the occurrence of a deviant tone. Neural activation was found within the triangular part of the inferior frontal cortex (BA45) (panel A, left arrow) and within superior frontal cortex (B).

Some of these activations were also seen in the analysis of the left hemisphere data. Fig. 4 shows an area of activation in the left hemisphere within the pars triangularis of the inferior frontal cortex (BA45). A second focus was found in the superior frontal cortex.

**Figure 5:**
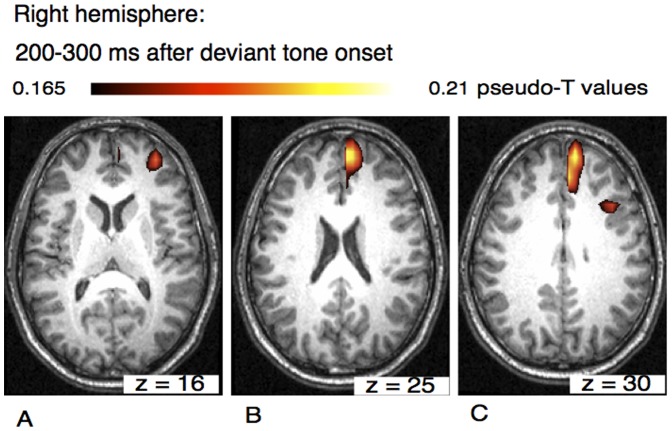
Axial view of the right hemisphere of the musical mismatch negativity within a time window of 200–300 ms after the occurrence of a deviant tone. Neural activation was found in superior frontal cortex (A), in medial frontal cortex (BA9) (B) and in medial and middle frontal gyrus (C).

In the later time window (200–300 ms after deviant tone onset) the beamformer analysis revealed activation in the right hemisphere within the frontal lobe in the superior frontal cortex, in the middle frontal and in the medial frontal cortices (Fig. 5).

**Figure 6:**
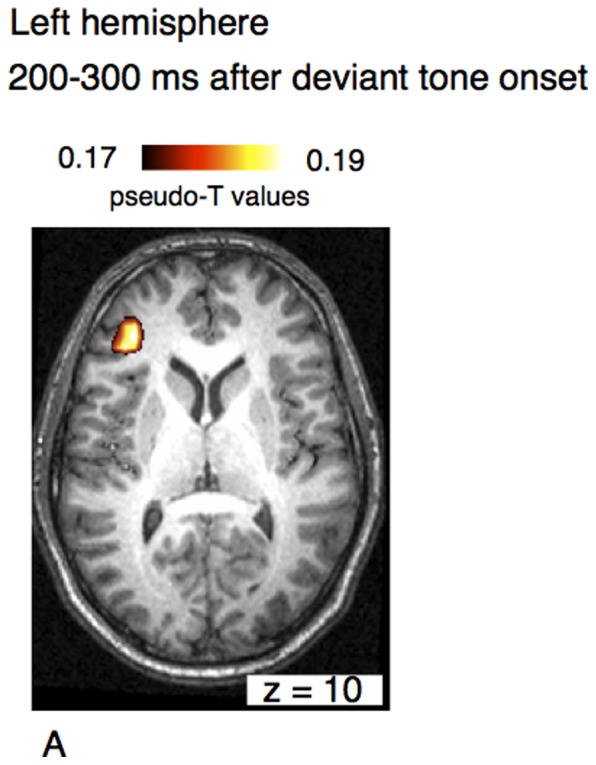
Axial view of of the left hemisphere of the musical mismatch negativity within a time window of 200–300 ms after the occurrence of a deviant tone. Neural activation was found within the inferior frontal cortex (A).

In the left hemisphere, neural activation within the time window between 200–300 ms was found in inferior frontal cortex. Axial views of these foci are shown in Figure 6.

Note that the sharp border of the medial frontal activation area results from the unilateral analysis which could reduce neural activation in the medial frontal areas. A whole head analysis, which we initially performed, yielded no significant activation. This negative result was presumably due to source correlations. We therefore performed another beamformer analysis only with frontal channels to check the stability of the analysis in the frontal part of the brain. The results show that both methods, a separate analysis of the hemispheres, and the analysis of frontal channels lead to similar activation patterns in frontal regions.

**Figure 7:**
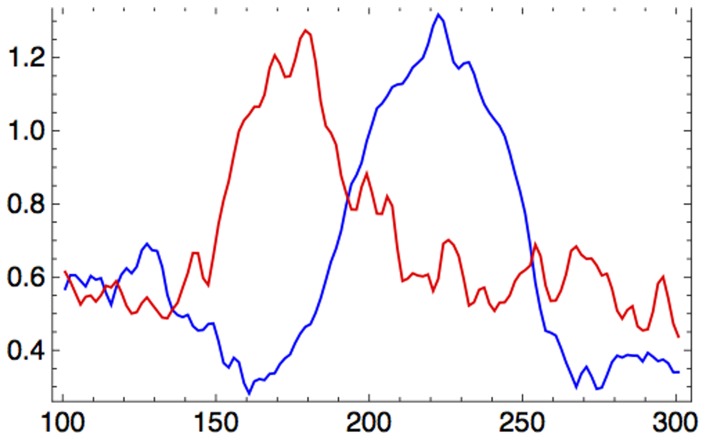
Group averages of the root mean square (RMS) virtual channels for STG (blue) and IFC (red) coordinates after the occurrence of a deviant tone within the MMN time window of 100–300 ms. The peak of IFC occurs slightly earlier than the peak of STG activation.

The time-course analysis (Fig. 7) of the whole trial within STG (blue line) and IFC (red line) revealed pronounced amplitudes in the time-window of the mismatch negativity. According to our analysis IFC activation appeared slightly earlier than STG activation.

## Discussion

Previous findings have demonstrated that temporal, and to a fewer extent, frontal cortical sources generate the auditory MMN elicited after a frequency or duration deviation within a repetitive tone series [23], [28]–[31]. These findings support the assumption that bottom-up as well as top-down processes are involved in the generation of this component. It has been proposed that the MMN arises within a hierarchically organized neural system, in which lower sensory areas are matched with models based on predictions from higher cortical areas, and that higher cortical areas themselves in turn adapt their model to the data received from lower cortical areas [54]–[56]. Consistent with this idea, our analysis revealed a distributed network of neural activation immediately after the occurrence of a deviant musical tone. The time-course analysis of the SAM data in our study showed that IFC peaked slightly earlier after the occurrence of the deviant tone than STG indicating that top-down processes are involved when errors are detected in well-known musical material.

In the time window between 100–200 ms after the onset of the tone deviation we found neural activation in the area of auditory cortices close to the superior temporal gyrus. This finding is in line with previous results based on dipole modeling as well as distributed source studies with EEG and MEG [16], [17], [57].

In addition we found activation in the pars opercularis of inferior temporal gyrus (Brodmann area 44, insula) and also in the left hemispheric Brodmann area 45. The left- hemisphere Brodmann area 45 (pars triangularis of the inferior frontal gyrus) constitutes together with the left-hemisphere Brodmann area 44 (pars opercularis of the inferior frontal gyrus) Broca's area, a center for speech. Language and music share a number of similarities. The structures of both domains unfold in time. The formation of words and chord progression are highly structured and predictable. Expectancy violations in both domains are therefore believed to be processed in overlapping neural generators [39], [58]. Whereas syntactic irregularities in language are processed in Broca's area [59], harmonic syntactic irregularities in music are presumably processed in the right hemispheric homologue of Broca's area [37], [38]. Activation in that region was also demonstrated in an fMRI study by Tillmann et al (2003), who compared neural activation between harmonically related and unrelated chords in a musical priming paradigm [60]. In that study, the BOLD signal in IFC (i.e. inferior frontal cortex, frontal operculum, insula) was stronger for unrelated than for harmonically related chords. Inferior frontal (BA44) activation with a right hemispheric asymmetry was also found by Koelsch et al. (2002) after the occurrence of a harmonic syntactically wrong chord [61].

However, activations in inferior frontal areas are generally believed to be associated with novelty processing and the detection of unexpected events. Incoming auditory information must be compared with stored information. Thus, our results may also suggest that activation in that area reflects neural processing of general auditory deviance detection. Moreover, the right inferior frontal cortex has been associated with attentional switching, in which the focus of attention is moved towards the deviant event [62]. Alternatively it has been speculated that, if a discrimination task is difficult, the IFC could help the superior temporal gyrus (STG) system to discriminate the stimuli linking the IFC to a contrast enhancement mechanism [31], [63]. Rinne et al. (2005) suggested that IFC activation could also be related to an inhibitory system allowing subjects to ignore stimuli when no reaction is necessary [18]. It was also proposed that the right inferior frontal gyrus could facilitate an attentional switch by inhibiting the previously attended object, therewith allowing to focus on a new stimulus [64]. Although the MMN could be elicited without the involvement of attention, studies have demonstrated effects of attention on the MMN [33], [34]. In ERP studies, attention switching is generally associated with the P3 component. The MMN is occasionally followed by a P3, but not necessarily [32]. Further investigation of the MMN-P3 relationship and IFC activation could therefore shed light on the role of IFC in attentional mechanisms.

It is possible that subjects learned the probabilities of the correct melody during the MEG session and used them to form predictions about the correct continuation of the melody that allowed the detection of a deviant. However, alternatively, or in addition, the activations we observed in the inferior frontal cortex may also be related to sensorimotor interactions. Learning to play a musical instrument results in a precise mapping between a musical note and the finger movement that is executed to produce a tone. Several studies have demonstrated interactions between the auditory and motor systems. Auditory-motor co-activation was investigated in an EEG study comparing a map-group, in which subjects learned to associate keypresses with musical sounds, with a non-map group, in which a random allocation of sounds to keypresses prevented such a learning. Auditory-motor co-activation was only observed in the map group [65]. In a similar fMRI study, non-musicians learned to play a melody on a keyboard and showed significant activation in Broca's area and the adjacent ventral pre-motor cortex (vPMC) after training when listening to the trained stimulus. This effect was not visible when participants listened to equally familar but motorically untrained melodies [66].

It is conceivable that in our study neurons of the vPMC, which is adjacent to Brodmann area 44, contributed to the auditory MMN. Our subjects had received piano training over the period of two weeks comprising 8 sessions each lasting 25 min. The piano training might have established an internal forward model linking a specific motor movement with an auditory sound [67]. It is conceivable that an internal forward model supported predictions about upcoming events and enabled a more precise estimate about upcoming tones. An internal model involving the motor system might help to detect easier auditory prediction violations, as manifested by the musical MMN.

Neural activation of IFC spread to the anterior insula. Increased neural activation in the area of the anterior insula has previously been observed during pitch discrimination [68] and melody perception [69]. Neural pathways have been demonstrated to connect the insula with the frontal operculum [70], auditory cortices (for an overview see: Bamiiou et al., 2003, [71]), as well as limbic structures. Accordingly, neural activation of the insula is associated with emotional processing and has been reported to correlate with pleasant musical experiences [72], but also in response to unpleasant music [73].

Within the early time window of 100–200 ms we found further neural activation in the orbitofrontal cortex (BA11). Recent fMRI studies have observed orbitofrontal activation in response to an unexpected chord at the end of a harmonically regular chord succession [39]. Neural activation following musical expectancy violation in the paralimbic system (orbitofrontal cortex and insula) could reflect affective experiences in music. The tone deviation at the end of the six-tone stimulus in our study might have been an unpleasant experience for the listener. OFC activation in response to unpleasant, but also pleasant music have also been shown in previous studies [72]–[74]. In addition to OFC further neural activation within the early time window of 100–200 ms was found in the frontal lobe in BA 10. The most anterior part of the frontal lobe is generally believed to be involved in complex cognitive processes like learning, reasoning, problem solving and memory.

Pitch is preferentially encoded on the right hemisphere [75], [76]. The results of the beamforming analysis seem to confirm this finding. More activation areas after the elicitation of the musical mismatch negativity were visible on the right.

Right IFC activation has been shown in some auditory mismatch studies using simple tone deviations [23]–[26], [28]–[31], [65]. Activation in that cortical area after an auditory deviation is therefore not confined to complex musical material. However, numerous fMRI, EEG as well as PET studies comprising auditory oddball paradigms did not find IFG activation and it has been argued that more complex stimuli could make it easier to find contributions of frontal areas [24]. This notion is supported by auditory mismatch studies showing that pitch deviants that are harder to detect elicit a stronger IFC activation than stimuli that are very easy to discriminate [31], [65]. Since IFC activation has not been found at all in MEG studies so far except for music and linguistic syntactic violations, it could be speculated that complex stimuli might enhance IFC activation.

Pronounced activation was also found in prefrontal cortex within the first 100 ms after tone onset. Our MEG stimulation was built up of a six-tone melody, in which the last tone was occasionally (20%) lowered by a minor third. The relatively long musical context in which the deviant tone occurred might have necessitated contributions of pre-frontal areas. It is also possible that explicit musical knowledge was needed to classify or integrate the deviation, which again in turn might have led to an activation of long-term memory systems in prefrontal areas. It has been shown in a study by Jacobson et al. (2005) with non speech stimuli that familiarity of context enhances the processing of auditory deviance detection [77]. Accordingly, long-term memory influences the processing of well-known auditory sequences. This is in line with auditory discrimination or musical training studies showing a stronger MMN after training [9]–[13]. Due to the prior sensorimotor training in our study, subjects were well familiar with the stimuli which is also in line with the aforementioned studies.

The time window between 200–300 ms revealed neural activation in the right hemisphere in superior frontal cortex and in medial and middle frontal cortex. The middle frontal gyrus is located between the superior and the inferior frontal sulci, rostral to the precentral gyrus. Direct anatomical connections have been demonstrated from auditory cortices to prefrontal areas including Brodmann area 9, the dorsal and ventral premotor cortex [73]. The results suggest that in the time window between 200–300 ms frontal contributions to the elicitation of the MMN increase especially in the right hemisphere. Prefrontal areas are associated with executive and cognitive functions like attention and working memory. In particular the medial frontal gyrus is associated with pre-response conflict, decision uncertainty, response error and negative feedback. It can be assumed that later stimulus processing involves higher order cognitive processes such as stimulus evaluation, memory retrieval, or decision making.

As mentioned earlier frontal generators in auditory MEG mismatch studies have only been found with linguistic stimuli so far [78], [79]. There is no evidence in the MMN literature for frontal activation in MEG with auditory oddball paradigms comprising frequency or temporal tone deviations. In an auditory mismatch study for example with spectrally rich tones and a combined measurement with EEG and MEG Rinne et al. (2000) did find STG and IFG activation in the EEG but only STG activation in MEG [17]. The authors speculated that the lack of evidence for frontal generators of the MMN in MEG studies could be the result of deeper lying frontal sources or their radial orientation which would make them invisible for MEG. The pronounced frontal and prefrontal activation in our MEG study is a new finding. It could be speculated that the beamformer is more than other methods sensitive to frontal neural signals allowing a more detailed picture of frontal activation. However, more studies have to be done to clarify this question.

## Conclusion

We localized the musically elicited MMN from MEG data with a beamformer. This method allows to analyze the generators of this component with high temporal resolution. The results suggest that immediately (100–200 ms) after the occurence of a deviant tone a distributed neural network is activated comprising auditory cortices, inferior frontal as well as pre-frontal areas. Moreover, a time-course analysis revealed that the MMN peaked slightly earlier within IFC as compared to STG. The musical context in which the MMN was presented might have initiated top-down mechanisms such as long-term memory where musical knowledge is stored, which might have contributed to classify or integrate the tone deviation.
